# An Unnecessary Evil

**Published:** 1899-10

**Authors:** H. H. Shapard


An Unnecessary Evil.—Very often we have patients present themselves to us whose teeth are literally ruined from the administration of iron compounds by physicians in the treatment of systemic troubles. This is entirely uncalled for; as the administration of iron, owing to the acidity and astringent property of many of these preparations, results in injury to the teeth upon which they act with great energy. They should be administered through a tube carried well back into the mouth, and preceded and followed with the use of an antiacid mouth-wash, such as carbonate of soda, a solution of ammonia, or milk of magnesia.

Physicians owe it to their patients to see that these precautions against the destruction of the teeth are carried out.

Why treat one ailment of the body and at the same time destroy the organs of mastication and bring on indigestion and other complications arising from the inability of the patient to properly masticate his or her food? Besides necessitating a big outlay of money for the services of a dentist, who, at best, cannot restore them to their original usefulness and beauty.—H. H. SHAPARD, D. D. S.

